# Leukocyte Trafficking in Cardiovascular Disease: Insights from Experimental Models

**DOI:** 10.1155/2017/9746169

**Published:** 2017-03-30

**Authors:** Daniel P. Jones, Harry D. True, Jyoti Patel

**Affiliations:** ^1^Division of Cardiovascular Medicine, Radcliffe Department of Medicine, British Heart Foundation Centre of Research Excellence, University of Oxford and John Radcliffe Hospital, Oxford OX3 9DU, UK; ^2^Wellcome Trust Centre for Human Genetics, Radcliffe Department of Medicine, University of Oxford, Oxford OX3 7BN, UK

## Abstract

Chemokine-induced leukocyte migration into the vessel wall is an early pathological event in the progression of atherosclerosis, the underlying cause of myocardial infarction. The immune-inflammatory response, mediated by both the innate and adaptive immune cells, is involved in the initiation, recruitment, and resolution phases of cardiovascular disease progression. Activation of leukocytes via inflammatory mediators such as chemokines, cytokines, and adhesion molecules is instrumental in these processes. In this review, we highlight leukocyte activation with the main focus being on the mechanisms of chemokine-mediated recruitment in atherosclerosis and the response postmyocardial infarction with key examples from experimental models of cardiovascular inflammation.

## 1. Atherosclerosis

Atherosclerosis is a chronic inflammatory condition affecting the medium- and large-sized arteries, characterised by the progressive development of lesions consisting of lipid, fibrosis, and inflammatory cell infiltrate within the tunica media [1]. The progression of these lesions into complex atherosclerotic plaques is in turn associated with vessel stenosis and plaque rupture with the generation of atheromatous thromboembolism [2]. It is the major pathological process underlying several cardiovascular diseases, such as myocardial infarction, and is thus a leading contributor to morbidity and mortality in the Western world [3].

The established link between hypercholesterolaemia and atherosclerosis led to the characterisation of atherosclerosis as primarily a disorder of lipids, a hypothesis that would dominate thinking for much of the 20th century. However, the last few decades have seen the establishment of inflammation as a key part of atherosclerosis and the important contribution of leukocytes to the initiation and progression of atherosclerotic plaques [1].

This section will focus on the role of leukocytes in atherosclerosis and specifically their migration and trafficking with a focus on the most recent developments in the field.

### 1.1. The Role of Leukocytes in Atherosclerosis

The macrophage is one of the defining cells present in an atherosclerotic lesion in addition to the macrophage-derived foam cell, an enlarged macrophage characterised by accumulation of oxLDL and cholesterol crystals [4]. Derived from circulating monocytes [5], the macrophage is a phagocytic cell that detects, processes, and clears pathogens and cell debris in addition to generating appropriate inflammatory responses.

Macrophage activation in atherosclerotic lesions is associated with the local release of inflammatory cytokines and reactive oxygen species, both of which contribute to the continued recruitment and activation of leukocytes. Activation is also associated with the release of plaque-destabilizing molecules such as matrix metalloproteinases [1]. Finally, upon transformation into a foam cell, the macrophage may undergo necrosis further releasing inflammatory stimuli and creating the necrotic core of advanced lesions [6].

Activation of macrophages is dependent upon ligand interactions with pattern recognition receptors [7]. In atherosclerosis, the study of a subset of these receptors, the toll-like receptors (TLRs), has allowed the key role of the macrophage to be demonstrated. In the atheromatous lesion, there is upregulation of TLR1, TLR2, TLR4, and TLR5 with oxLDL (a constituent component of atheromatous lesions) specifically being shown to upregulate TLR4 mRNA levels in vitro [8]. Deletion of TLR4 in ApoE^−/−^ (apolipoprotein E knockout) mice leads to significantly marked reductions in lesion formation [9], and this is also seen in TLR2^−/−^ApoE^−/−^ mice [10], whilst ApoE^−/−^ mice with inactivating mutations of M-CSF (macrophage colony stimulating factor) also show marked reductions in both lesion size and macrophage content [11].

Lymphocytes, particularly T-lymphocytes, are also present in atherosclerotic lesions albeit in smaller numbers than macrophages [12]. However, their role is important as highlighted in lymphocyte-deficient animal models of atherosclerosis displaying reduced lesion burden [13]. Lymphocytes are recruited into the subendothelial space in a similar manner to monocytes and subsequently become activated by locally present cytokines, such as IL-12 and IL-18 [13]. Clonal expansion of T-cells and a dependence on CD40 ligand has suggested that T-cell activation can also occur due to antigen recognition, most likely an auto-antigen present in the atheroma [14].

In turn, T-cell activation plays a role in atherosclerosis with interferon-gamma, the signature cytokine of T helper 1 (TH1) cells, being present in human atherosclerotic lesions and in turn being associated with enhanced cytokine activity, increased macrophage activation, and reduced collagen formation [14]. The role of other CD4+ T-cell types as well as CD8+ T-cells is less clear, whilst B-cells appear to play an athero-protective role [15]. B-cells are present in plaques with B-cell transfer in immune-deficient mice or administration of anti-oxLDL antibody reducing lesion burden, although no correlation between antibody titres and atherosclerosis has been found in humans [15].

### 1.2. Initiation: The Recruitment of Leukocytes in Atherosclerosis

Atherosclerosis occurs at specific sites in the arterial tree where turbulent flow is present, such as the aortic root and arch, in humans as well as animal models of atherosclerosis with “fatty streak” lesions present from the first decade of life in humans [2]. Whilst numerous pathological observations demonstrate the capacity of endothelial denudation to cause lesion formation, it is rather endothelial dysfunction thought to be critical in the initiation of atherosclerosis [16].

**Figure 1:**
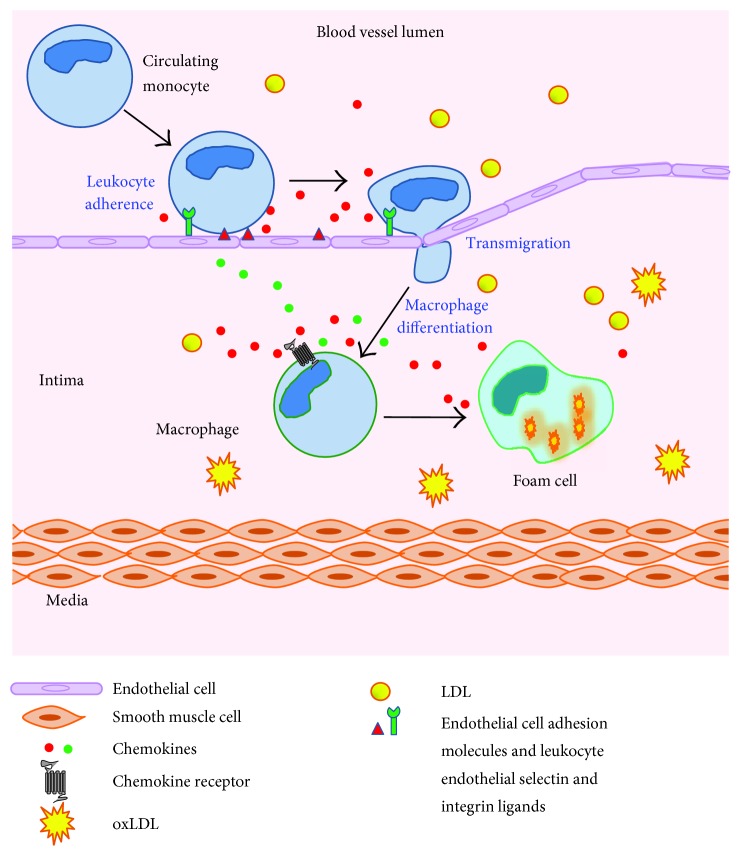
The formation of atherosclerotic plaques is characterised by the recruitment of monocytes to the artery wall directed by chemokines, followed by adherence and transmigration across the endothelium. The accumulation of lipid deposits containing oxLDL in the intima is then taken up by plaque macrophages to form foam cells resulting in fatty streak lesion formation.

Further to this, our understanding of vascular biology has come to demonstrate the important interactions of endothelial cells in trafficking leukocytes to areas of local inflammation and injury. Indeed, the expression of adhesion molecules on their surface allows subsequent interaction between those molecules and leukocyte selectins to enable rolling and extravasation [16] (Figure 1). As a result, normal endothelium resists leukocyte adherence whilst dysfunctional or activated endothelium demonstrates firm leukocyte attachment.

The expression of several proteins in endothelial cells is linked to the levels of shear stress. Early *en-face* immunohistochemical staining in animal studies of atherosclerosis demonstrates the upregulation of VCAM-1 (vascular cell adhesion molecule-1) in turbulent flow sites in hyperlipidaemic animals compared to wild-type animals [17]. In addition, ApoE^−/−^ mice with hypomorphic variants of VCAM-1 display reduced lesion formation [18]. Concurrently, ICAM-1 (intracellular adhesion molecule 1) is upregulated in these sites; however, this upregulation also occurs in control animals [17]. Both VCAM-1 and ICAM-1 serve to recruit leukocytes to the endothelial surface.

Conversely, in sites of laminar flow, there is upregulation of several “athero-protective” genes such as eNOS (endothelial nitric oxide synthase), with the subsequent nitric oxide release serving to combat oxidative stress as well as to directly limit VCAM-1 expression through the inhibition of NF-κB [19]. Indeed, the athero-protective role of eNOS is demonstrated by the ApoE^−/−^eNOS^−/−^ mouse which shows increased lesion burden, VCAM-1 expression, and plaque macrophage content [20]. Conversely, the overexpression of eNOS in a ApoE^−/−^ mouse, in conjunction with the overexpression of the rate limiting enzyme (GTPCH1) which is involved in its cofactor synthesis, to prevent eNOS uncoupling, shows markedly reduced lesion burden [21].

Thus, it appears that in endothelial sites of turbulent flow, there is upregulation of adhesion molecules with enhanced upregulation in the presence of hyperlipidaemia and relative downregulation of endothelial genes important in preventing atherogenesis. This initial process and the subsequent recruitment of leukocytes as well as lipid into the subendothelial spaces of turbulent flow sites lead to the creation of a local inflammatory environment, where lipid is oxidised and leukocytes are activated, which is critical in the development of lesions [1].

### 1.3. Leukocyte Migration and Retention

At the sites of inflammation, leukocyte attraction and retention not only requires the local expression of endothelial adhesion molecules but also the activation of leukocytes and subsequent conversion into a high affinity state. This is typically achieved through the local release of chemical mediators, principally the chemokines (chemotactic cytokines), from local tissue-resident cells [22].

In the initial lesion, the entrapment of LDL (low density lipoprotein) and its subsequent oxidation leads to localised inflammation and the release of chemokines with other inflammatory mediators; this synergises with shear stress-induced expression of adhesion molecules to recruit leukocytes which become activated by and then contribute to the inflammatory environment [1].

Chemokines are 8–12 kDa proteins that signal through their respective G-protein-coupled receptors (GPCRs). They are released from endothelial cells, platelets, macrophages, and lymphocytes following activation by inflammation and cardinal inflammatory mediators, such as TNF-*α*. Upon release, chemokines bind to glycosaminoglycans on cell surfaces to establish local gradients that are able to activate and then direct leukocytes to specific sites [15].

Chemokines and chemokine receptors are significantly implicated in atherosclerosis with chemokine receptors known to be expressed within animal and human plaques and their expression being correlated to progression [23].

Although the chemokine system displays a great deal of redundancy, the development of chemokine receptor knockouts in atherosclerosis murine models has given insight into their role. This was first demonstrated in the ApoE^−/−^CCR2^−/−^ murine model which shows significant reductions in macrophage content and plaque content compared to ApoE^−/−^ mice [24]. Additionally, the knockout of CCR5 in ApoE^−/−^ displays striking reductions of at least 50% of lesion area as well as greater reductions in macrophage content and an increase in smooth muscle content [25].

In vivo antichemokine strategies have also been successful in reducing plaque burden. The antagonism of RANTES (regulated upon activation, normal T-cell expressed and secreted) with met-RANTES in LDLR^−/−^ (LDL receptor) mice for 14 weeks resulted in significant lesion area reduction as well as a reduction in CCR2 expression suggesting an anti-inflammatory and antiatherogenic effect [26]. The use of both adenoviral and lentiviral vectors to express vCCI (viral chemokine inhibitor), a broad-spectrum chemokine inhibitor, also produces a marked antiatherogenic effect in ApoE^−/−^ mice [27].

However, chemokines do not appear to be universally proatherogenic, with the ApoE^−/−^CCR1^−/−^ murine model demonstrating an increase in lesion area with increased levels of T-cell infiltration, providing further evidence of the contribution of T-lymphocytes [25].

Chemokines are also known to play an apparent role in the retention of leukocytes within the plaque, although this has not been as comprehensively studied as the process of leukocyte recruitment in atherosclerosis. For example, the chemokine CX3CL1 promotes macrophage adhesion to smooth muscle cells (SMCs) within coronary artery plaques and its expression is elevated in hyperlipidaemia. Conversely, a reduction in CX3CL1 expression reduces macrophage adherence to SMCs [28].

Perhaps most striking is the apparent role of chemokines in leukocyte migration in regression models of atherosclerosis. In the surgical model, whereby regression in an affected artery is induced by transfer to a normo-lipidaemic animal, there is a reduction in VCAM-1 levels in the atherosclerotic artery but, conversely, an increase in CCR7 levels. Further to this, when recipient animals were treated with antibodies against the CCR7 ligands, the regressing lesions were markedly impaired, with retention of leukocyte content seen as well, suggesting a failure of migration [29].

Finally, in addition to the principal role of chemokines in leukocyte migration in atherosclerosis, other molecules such as chemotaxins may also play a role. Leukocyte cell-derived chemotaxin 2 has recently been shown to correlate with atherosclerotic lesion burden and macrophage content in human tissue samples [30], and further to this, LECT-2 treatment of human umbilical vein endothelial cell culture has been shown to produce c-Jun N-terminal kinase-mediated increases in ICAM-1 and MCP-1 expressions [31] which would both serve to enhance leukocyte recruitment. Further in vivo studies with LECT-2 and other chemotaxins may well demonstrate important contributions to this aspect of atherosclerosis.

### 1.4. Leukocyte Tracking in Experimental Models of Cardiovascular Disease

One of the more recent developments in atherosclerosis research has been the production of murine models that facilitate tracking of myeloid cells thus providing new insights into myeloid cell biology beyond the work previously discussed regarding the role of chemokines and shear stress-linked regulation of genes.

It has been established, for instance, that the macrophages and foam cells in murine experimental plaques are mainly recruited from circulating monocytes as opposed to local resident cells [5]. An example is seen in a murine model expressing an alternative isoform of CD45, which after full engraftment of a bone marrow transplant demonstrates a dominance of donor myeloid cells in plaques induced by arterial injury [5]. A further example is seen with the adoptive transfer of radioactively labelled monocytes into ApoE^−/−^ animals which demonstrates a dominance of donor myeloid cells in the plaque [32].

It has also been possible to elucidate the role of different macrophage subtypes. The creation of a murine model expressing GFP under the CX3CR1 promoter revealed the existence of low-expression and high-expression populations with expression of CX3CR1 being negatively correlated to the expression of Ly6C and Gr-1. It was subsequently found that CX3CR1^lo^/Ly6C^hi^ monocytes preferentially migrate to the sites of acute inflammation whereas the CX3CR1^hi^/Ly6C^lo^ population does not [33].

Further to this, it is now understood that the peripheral blood monocytosis that develops in ApoE^−/−^ mice, when induced through diet to develop hypercholesterolaemia, is dominated by the CX3CR1^lo^/Ly6C^hi^ population. Indeed, in this model, the CX3CR1^lo^/Ly6C^hi^ population doubled every month on a high-fat diet and is correlated with lipid levels; conversely, the monocytosis of this population is attenuated by statin treatment [34]. Additionally, recent data utilising spleen transplantation experiments has highlighted the role of the spleen as an additional source of CX3CR1^lo^/Ly6C^hi^ monocytes proving not only to be a reservoir but also an active source of monocyte generation [35].

This work has also been applied to further demonstrate the role of chemokines in leukocyte migration in atherosclerosis. The monocytosis of the CX3CR1^lo^/Ly6C^hi^ population is not seen in animals on high-fat diet when CCR2 is knocked out for instance [36]. In addition, the use of radioactive bead labelling in different monocyte subsets in a CCR2 null murine model that sees an atherosclerotic aortic arch transplanted into it demonstrated the dependence of CX3CR1^lo^/Ly6C^hi^ monocytes on CCR2 for migration [37].

It has also offered further insights into the role of adhesion molecules. For example, the CX3CR1^lo^/Ly6C^hi^ monocyte population is now known to express higher levels of PSGL-1 (P-selectin glycoprotein ligand-1), an adhesion molecule, compared to CX3CR1^hi^/Ly6C^lo^ monocytes. In turn, the PSGL-1 null mouse displays a reduced migration of CX3CR1^lo^/Ly6C^hi^ monocytes to atherosclerotic plaques with a concordant reduction in lesion area [38].

Finally, a murine model expressing GFP under the control of the human CD68 promoter has recently been created which drives GFP expression in all monocytes of the blood, spleen, and bone marrow. Most promisingly, these monocytes retain high levels of GFP expression for at least 72 hours after differentiation into macrophages and so, unlike the CX3CR1^GFP^ model previously discussed, enables the study of monocyte fate as well as initial trafficking and recruitment [39].

Indeed, this model has already been utilised in atherosclerosis research with the generation of CD68^GFP^ ApoE^−/−^ mice, where the expression of GFP had no significant effect on the atherosclerosis process and allowed clear visualisation of plaque macrophages, with quantification by GFP correlating with traditional macrophage quantification methods [40]. Adoptive transfer of bone marrow monocytes into ApoE^−/−^ with established plaques demonstrated clear visualisation of GFP^+^ cells 72 hours posttransfer further confirming the ongoing recruitment of peripheral monocytes as the source of the plaque macrophage population [40]. In contrast to traditional methods of assessing monocyte kinetics and fate, this model appears to not profoundly alter plaque biology and thus promises to offer new and exciting insights in the field. New experimental models of cardiovascular disease not only provide a better understanding of the processes behind leukocyte recruitment and retention but also enable the discovery of novel therapeutic targets in cardiovascular inflammation.

## 2. Myocardial Infarction and Heart Failure

According to the American Heart Association, myocardial infarction (MI) and congestive heart failure (CHF) mortalities are the major contributors of the cardiovascular diseases that are the leading causes of death in the Western world [41]. MI describes the death of cardiac tissue due to prolonged ischaemia (20 minutes plus) or through the occlusion of a coronary vessel commonly by lumen-reducing atherosclerotic plaques and subsequent thrombosis formation [42].

Factors that increase the lifetime risk of an acute (i.e., MI) or chronic (CHF) coronary syndrome, which manifest over decades, are a high-lipid diet (atherosclerosis), low levels of exercise, chronic diseases (diabetes), smoking, and genetic predisposition (familial hypercholesterolaemia) [43]. The average age for the first MI is 65 years for women and 56 years for men, of which an increasing number of patients survive post-MI due to the improvements in reperfusion techniques and acute coronary care [44]. However, surviving patients are faced with restricting morbidities, an increased mortality risk of 8–12% [45] and a further ischaemic event risk of 1 in every 2, both within the first year [46]. Clinical management post-MI is improving, but the mechanisms leading to heart failure and the possibility for prevention offer a therapeutic target for cardiovascular disease research.

### 2.1. Myocardial Infarction

Cardiovascular disease was first described in 1772 by Herberden [47]; symptoms of breast pain were later diagnosed as angina pectoris (the temporary reduction in blood flow to the heart causing chest pain after exercise) with leukocytosis documented in fibrosed left ventricles of prolonged angina mortalities. However, MI was not identified until 1912 [48], once the relationship between coronary artery disease, acute/prolonged angina, and coronary thrombosis had been established, via the advent of echocardiography (ECG) in defining the pathological conditions. Since then, scientists have attempted to elucidate the mechanisms that underlie MI, that is, the potential role of leukocytes and inflammation. However, in the clinic, emergency reperfusion by fibrolytic agents targeting the thrombosis was and remains the staple treatment post-MI for the survival of patients [49], which can result in adverse left ventricle remodelling and heart failure.

The most frequent coronary vessel occluded in MI is the left anterior descending artery (LAD) which nourishes the left ventricle (LV), and the ischaemia that follows impairs the endothelial vascular integrity leading to leukocyte infiltration [50]. The cardiomyocytes, tissue-resident macrophages, and fibroblasts, deprived of nutrients, undergo necrosis within the first 30 minutes [51], passively releasing damage-associated molecular patterns (DAMPs), such as ATP and hyaluronic acid by breakdown of the extracellular matrix (ECM). TLRs 2 and 4 on local macrophages and neutrophils are activated by these DAMPS and stimulate a proinflammatory immune response via chemokines, cytokines, and vascular adhesion molecules, through intracellular MAPK (mitogen-activated protein kinases) and NF-κB signalling [51]. The tissue macrophage release of CXC chemokines, TNF-*α* (activating endothelial cells and increasing vessel permeability), and IL-1*β*/IL-18 stimulates neutrophil recruitment within 12–24 hours post-MI. The positive self-feedback loop of CCL8 secretion recruits further neutrophils to the infarct for the phagocytosis of necrotic, autophagic, or apoptosed cardiac tissue [51]. Neutrophils not only release MMPs to breakdown the ECM and the cardiomyocytes within the infarct but also produce soluble IL-6 which activates endothelial cells to express chemokine ligand CCL2 and VCAM1 for monocyte recruitment [52]. Neutrophil clearance (at around day 7) manifests via high concentrations of the aforementioned proinflammatory chemokines and the release of death signals, for inhibition of neutrophil migration/prophagocytosis, such as lactoferrin and annexin A1 [53].

### 2.2. The Biphasic Immune Response to MI

**Figure 2:**
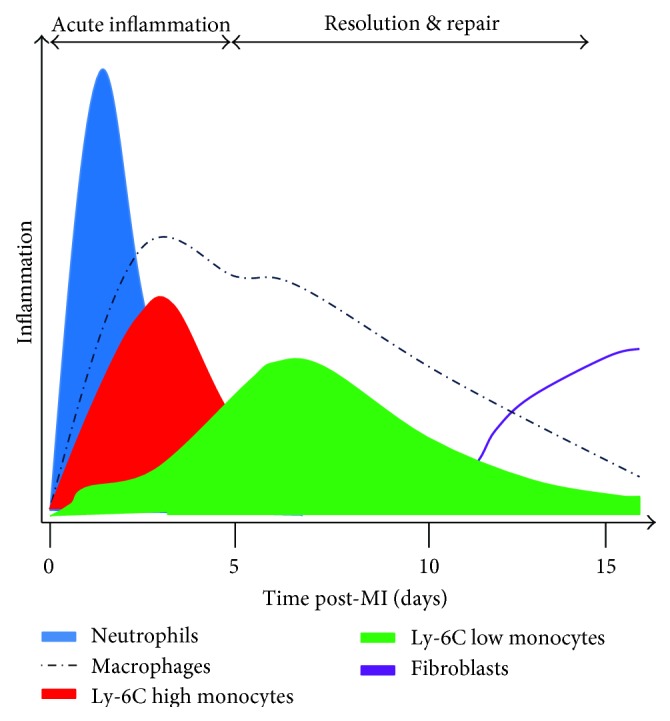
Track of innate immune system inflammatory cell variance over the time course of 15 days within the ischaemic left ventricle, postmyocardial infarction in mice.

The immune response to acute MI can be divided between the inflammatory phase at days 1–4 and the healing phase from days 5–14 from which point the scar matures [54] (Figure 2). Certain monocytes dominate these different phases and are masters of the adverse LV remodelling and heart failure post-MI as highlighted by Nahrendorf et al. [55]. This key study identified two subsets of monocytes that were alternatively “recruited” throughout MI inflammation and healing, with different but complementary functions: proinflammatory Ly6C^hi^ monocytes (recruited via CCR2) and the prohealing Ly6C^lo^ monocytes (recruited via CX3CR1), differentiating to various subsets of macrophages within the classically activated M1 and the alternatively activated M2 phenotypes [56]. In humans, the proinflammatory monocytes express CD14++CD16− and the prohealing monocytes express CD14+CD16++, resembling their murine counterparts and allowing the study of MI in mice to simulate the human response [57].

With the innate immune response underway, tissue-resident macrophages are proliferating under the control of M-CSF, from activated endothelial cells, fibroblasts, and macrophages, and secrete increasing levels of CCL2. CCL2 stimulates the recruitment of the Ly6C^hi^ monocytes from the blood pool via their receptor CCR2 and initiates HSC (haematopoietic stem cell) progenitor proliferation in the bone marrow [58].

Furthermore, MI activates the neuro-humoral axis via sympathetic innervations of adrenaline secretion for *β*3-adrenergic receptors in the bone marrow, reducing levels of CXCL12 (the “monocyte progenitor egress” inhibitory cytokine) and allowing HSC relocation to the spleen for proliferation and maturation [54]. Limiting the immediate fibroblast-mediated ECM remodelling and infarct healing is the result of M1 macrophages NLRP3 inflammasome activation (releasing and converting pro-IL-1B to IL-1B by caspase-1) via M1 engulfing of cholesterol crystals, promoting fibroblast proteolytic enzyme release to assist in the ECM degradation [59]. Splenic monocyte recruitment is activated via angiotensin II signalling, with increased levels due to the release of ACE (angiotensin-converting enzyme) from dead cardiac monocytes, suggesting the benefit of post-MI therapy via ACE inhibitors to reduce the size and leukocyte populations in the infarct [60].

Extravasation of Ly6C^hi^ monocytes to the infarct continues the acute phase response and the efferocytosis of dead cardiac tissue including the apoptotic neutrophils, preparing the infarct site for new ECM and collagen deposition by fibroblasts and Ly6C^lo^ monocytes. Prolonged inflammation via the Ly6C^hi^ monocytes is associated with dilative remodelling (LV thinning) and systolic dysfunction, ultimately leading to heart failure when the inflammatory phase is not resolved and Ly6C^hi^ cells repopulate the LV weeks to months postinfarct healing [61].

The mechanisms of monocyte recruitment in MI were first challenged by Kaikita et al. using CCR2^−/−^ mice [62]. The authors noted a great impairment of macrophage accumulation in the infarcted region within 7 days post-MI and a critical reduction in LV remodelling. At the time, they were unaware of the biphasic immune response but they concluded on the importance of CCR2 for monocyte-macrophage recruitment, suggesting the remainder of monocytes unaffected by CCR2^−/−^ could be due to CCR1 and CCR5 low affinity for CCL2 released by the infarct.

Further studies have shown that a balance between proinflammation versus healing is key for cardiac remodelling as CCR2^−/−^ animals have impaired necrotic cell clearance, collagen deposition, and angiogenesis with the predisposition for cardiac rupture and immediate death [63]. Overactivation of the healing phase leads to concentric remodelling and diastolic dysfunction with LV stiffness (predisposing heart failure) [64].

Building on the work of Hanna et al. [65], which described the orphan nuclear hormone receptor, Nr4a1, as a vital regulator of monocyte development in the bone marrow affecting the levels of Ly6C^lo^ monocyte populations, with decreased peripheral levels compared to the Ly6C^hi^ populations (in Nr4a1^−/−^), Hilgendorf et al. [66] identified that the recruitment of the prohealing, blood-patrolling, Ly6C^lo^ monocytes is amplified by a Ly6C^hi^ monocyte to Ly6C^lo^ macrophage conversion, with Nr4a1 levels fluctuating between the two cell phenotypes with high levels dampening the proinflammatory macrophage secretions.

The previous finding that ACE inhibitors reduced the leukocyte populations can now be explained by not just inhibiting Ly6C^hi^ monocyte recruitment but its subsequent differentiation to the infarct macrophage populations. Additionally, Ly6C^lo^ monocytes are also recruited to the infarct from the blood via CX3CL1 release through endothelial cells [56]. With the accumulation of dead neutrophils, inflammatory resolution is stimulated by neutrophil “eat me” signals (phosphatidylserine) as well as their expression of lipoxins and resolvins, engaging the M2 phenotype conversion [67]. Phagocytosis of these neutrophils by macrophages encourages the expression and secretion of IL-10, the anti-inflammatory cytokine, expressed in greater proportions from the Ly6C^lo^ cells as well as TGF-*β*, a stimulator for ECM gene expression by cardiac myofibroblasts. Angiogenic factors are also secreted by the Ly6C^lo^ cells such as VEGF (vascular endothelial growth factor) to restore oxygen supply by neovascularisation and to establish the granulation tissue for scar formation/maturation [57]. Noncontractile scar formation is the result of myofibroblast-depositing collagen following activation via TGF-*β* (transforming growth factor beta) signalling and TCRP6 (transient receptor potential 6) expression to switch from cardiac fibroblasts [68].

## 3. Concluding Remarks

From a clinical perspective of leukocyte migration in atherosclerosis, the chemokine system represents a particularly excellent candidate for exploring therapeutic options. Whilst the chemokine system displays a great deal of redundancy, there are clear and powerful effects seen in specific murine knockouts of key chemokine receptors which might be replicated. Indeed, the chemokine system could be targeted through several different avenues, such as classical GPCR antagonists, for example. Due to the important role of chemokines in wound healing and infection control, long-term therapy would likely be associated with adverse effects. However, utilising chemokine inhibition to stabilise atherosclerotic plaques in particular circumstances could well provide clinical benefit. Such examples might include delivery of therapy to stabilise plaques following myocardial infarction to prevent further rupture or vessel stenosis or delivery of therapeutics in transient ischaemic attacks to reduce stroke risk due to carotid artery atherosclerosis. With several drug targets against chemokines already identified in drug discovery programmes, this field bears considerable future potential [69]. In addition, the major targets for MI and heart failure research are to regulate the inflammatory monocyte infiltration phase and to reduce the potential for adverse remodelling and future heart failure. Given that the clinical trials of anti-inflammatory medications are failing (anti-TNF/IL-1*β*) [70], the mechanisms that underlie adverse and normal MI healing are yet to be uncovered.

## Conflicts of Interest

The authors declare that they have no conflicts of interest.
